# Establishment and application of a novel method based on single nucleotide polymorphism analysis for detecting β-globin gene cluster deletions

**DOI:** 10.1038/s41598-020-75507-6

**Published:** 2020-10-26

**Authors:** Siqi Hu, Wenli Zhan, Jicheng Wang, Jia Xie, Weiping Zhou, Xiaohan Yang, Yukun Zeng, Tingting Hu, Lei Duan, Keyi Chen, Li Du, Aihua Yin, Mingyong Luo

**Affiliations:** 1 Medical Genetic Centre, Guangdong Women and Children’s Hospital, Guangzhou Medical University, 521-523 Xingnan Avenue, Panyu District, Guangzhou, 511400 China; 2 Medical Genetic Centre, Guangdong Women and Children Hospital, Guangzhou, China; 3 Medical Genetics Center, Affiliated Shenzhen Maternity & Child Healthcare Hospital, Southern Medical University, Shenzhen, Guangdong China

**Keywords:** DNA, Mutation, Molecular biology

## Abstract

β-Globin gene mutations reduce or terminate the production of beta globin chains, of which approximately 10% are large deletions within the β-globin gene cluster. Because gene deletion leads to loss of heterozygosity at single nucleotide polymorphism (SNP), a novel method for detecting β-globin gene cluster deletions based on SNP heterozygosity analysis was established in this study. The location range of SNPs was selected according to the breakpoint of β-globin gene cluster deletions. SNPs were screened using bioinformatics analysis and population sequencing data. A novel method which enables genotyping of multiplex SNPs based on tetra-primer ARMS-PCR was designed and optimized. Forty clinical samples were tested in parallel by this method and MLPA to verify the performance of this method for detecting β-globin gene cluster deletion. Six informative SNPs were obtained, achieving heterozygote coverage of 93.3% in normal individuals. Genotyping of six SNPs were successfully integrated into two multiplex tetra-primer ARMS-PCR reactions. The sensitivity, specificity, positive predictive value and negative predictive value of the method for detecting β-globin gene cluster deletion were 100%, 96.30%, 92.86%, and 100%, respectively. This is a simple, cost-effective and novel method for detecting β-globin gene cluster deletions, which may be suitable for use in combination with MLPA for thalassemia molecular testing.

## Introduction

Thalassemias are a group of inherited autosomal recessive hematologic disorders that cause hemolytic anemia via disrupted globin chain synthesis^1^. As one of the most common genetic diseases worldwide, it is prevalent in the Mediterranean, Middle East, central Asia, India, and southern China^2^, with an estimated 1–5% of the global population carrying the thalassemia trait^3^. Thalassemias are broadly characterized as α or β-thalassemias, the clinical symptoms of which may vary from none to severe, depending on the type^2^. Thalassemia major, a crippling and fatal disease which seriously damages human health, is considered an important medical and public health risk.

Beta globin gene cluster mutations reduce or inhibit production of beta globin chains. Over 300 such mutations have been reported so far. Although the vast majority are point mutations, approximately 10% are due to large deletions within the β-globin gene cluster causing β^0^-thal, (δβ)^0^-thal, ^G^γ(^A^γδβ)^0^-thal, and hereditary persistence of fetal hemoglobin (HPFH) (https://globin.bx.psu.edu/hbvar/)^4^. Eleven types of β-globin gene cluster deletions have been reported in the Chinese (Fig. 1.1-A, Table 1)^5–10^. Coinheritance of these deletions with other β-thalassemias or Hb variants results in phenotypes ranging from asymptomatic to β-thalassemias major^11^. Therefore, it was felt that more attention should be paid to the detection of beta globin gene cluster deletion in regions with a high prevalence of thalassemia.

**Figure 1:**
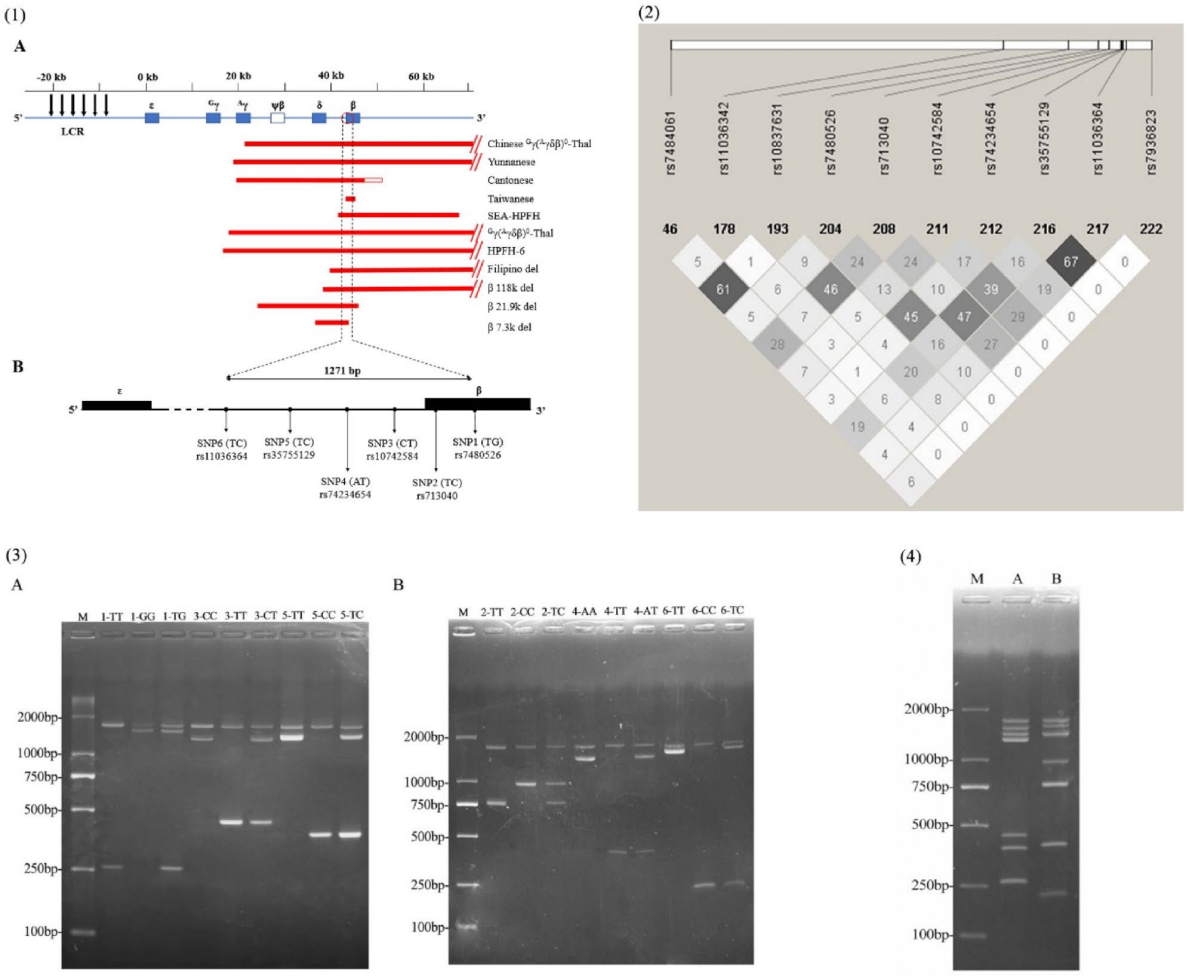
(1) The deletion ranges of eleven β-globin gene cluster deletions in Chinese (**A**); and the locations of the panel of six SNPs (**B**). (2) Ten candidate SNPs were obtained following bioinformatics analysis. (3) Establishment of the single SNP genotyping system by tetra-primer ARMS-PCR. (4) Establishment of the multiplex SNPs genotyping system by two tetra-primer ARMS-PCR reactions.

**Table 1:** Eleven β-globin gene cluster deletions in Chinese.

Type	HGVS name	Gene deletion	
Chinese ^G^γ(^A^γδβ)^0^-Thal	NC_000011.9:g.5191148_5270051del	78,904 bp	Ggamma1 to beta gene
Yunnanese	NC_000011.9:g.5204076_5271203del	67,128 bp	Ggamma1 to beta gene
Cantonese	NC_000011.9:g.5240000 ~ 5246696_5271087del	31,088 bp	Ggamma1 to beta gene
Taiwanese	NC_000011.9:g.5247493_5248849del	1357 bp	Beta gene
(SEA)-HPFH	NC_000011.9:g.5222878_5250288del	27,411 bp	Beta gene
^G^γ(^A^γδβ)^0^-Thal	NC_000011.9:g.5179272_5271683del	92,412 bp	Ggamma1 to beta gene
HPFH-6	NC_000011.9:g. 5193974_5273251del	79,278 bp	Ggamma1 to beta gene
Filipino del	NC_000011.9:g.5134113_5252589del	118 kb	Beta gene
β118k del	NC_000011.9:g.5135464_5254173del	118 kb	Delta to beta gene
β21.9 k del	NC_000011.9:g.5246909_5268823del	21.9 kb	HBBP1 to beta gene
β7.3 k del	NC_000011.9:g.5247824_5255222del	7.3 kb	Delta to beta gene

Initial screening for thalassemia via red blood-cell indices and hemoglobin analyses, is followed by genetic testing to further identify the specific mutations underlying β-thalassemia in DNA^12^. Molecular tests, such as gap-polymerase chain reaction (Gap-PCR), Southern blot analysis, and multiplex ligation dependent probe amplification (MLPA), are commonly used to detect β-globin gene cluster deletions^13^. However, Gap-PCR is only capable of detecting those deletions for which the breakpoints are known, wherein specific primers need to be designed for each gene deletion type and negative results do not rule out other types of gene deletions^14^. Southern blot analysis is both time consuming and technically demanding, and success is very much dependent upon the availability of hybridization probes^13^. MLPA, an in vitro diagnostic tool, is also not suitable for primary clinical laboratory due to its technical complexity, high cost and the specialized equipment required^15^. In view of the limitations of the current method, a simple, economical and low-tech method is needed to detect the deletion of β-globin gene cluster.

Single nucleotide polymorphisms (SNPs) are the most common type of genetic variations, and are widespread in the human genome^16^. As third generation of molecular markers, SNPs are utilized to reveal evolutionary histories and common genetic polymorphisms that account for heritable risks for common diseases^17,18^. Loss of heterozygosity (LOH) is the most frequent manner by which a mutant allele is lost in human cancers. LOH occurs through a gross chromosomal event that results in loss of an entire gene and the surrounding chromosomal region^19^. When LOH occurs, it presents as a single copy which cannot be heterozygous at a SNP location and therefore the region shows loss of heterozygosity. Based on LOH principles, we established a method that enables the detection of β-globin gene cluster deletion using heterozygosity analyses of SNPs. Many methods have been developed for SNP genotyping over the past several years^20^. These include methods such as SNaPshot approach (Applied Biosystems, CA)^21^, pyrosequencing that relies on DNA sequencing^22^, and TaqMan system (Applied Biosystems, CA)^23^, as well as Dynamic allele-specific hybridization (DASH; DynaMetrix, UK)^24^ which is based on hybridization assays. These methods were developed for use with high throughput techniques but costs and practicability have so far limited the wider application of these technologies. The tetra-primer amplification refractory mutation system PCR (tetra-primer ARMS–PCR) developed by Ye et al. has proved to be an economical and effective method for SNP genotyping^25–27^. Positioning the two outer primers at different distances from the polymorphic nucleotide, causes the two allele-specific amplicons differ in length, allowing them to be discriminated by gel.

The current study developed a novel method for detecting β-globin gene cluster deletion based on heterozygosity analyses of SNPs and established a genotyping system for SNPs using tetra-primer ARMS-PCR technology. It detected deletional types of β-globin gene cluster in a more convenient and cost-effective manner, and showed potential as a routine laboratory application, which provided an alternative approach to preliminary screening and clinical detection of thalassemia.

## Results

### A panel of SNPs

First, data containing 233 SNPs (Southern China) were obtained from the 1000 Genomes Project corresponding to the location of the SNPs region selected above. Next, 10 candidate SNPs were obtained following MAF, HWE and LD tests as follows: rs7484061, rs11036342, rs10837631, rs7480526, rs713040, rs10742584, rs74234654, rs3575512, rs11036364, and rs7936823, respectively (Fig. 1.2). Population allele frequencies of the candidate SNPs were subsequently identified in 105 normal individuals (Supplementary Table S1). Finally, a panel of 6 informative SNPs was obtained as follows: rs7480526, rs713040, rs10742584, rs74234654, rs35755129, and rs11036364, respectively (Table 2). This indicated that 98 out of 105 samples carried at least one heterozygous SNP, revealing a heterozygote coverage of 93.3% (95% CI 88.57–97.14%) in normal individuals that was attributable to the panel of six SNPs.

**Table 2:** The panel of six informative SNPs employed in this study.

No	SNP ID	HGVS	MAF (Southern China)
SNP1	rs7480526	NG_000007.3:g.71113T>G	0.181 (G)
SNP2	rs713040	NG_000007.3:g.70603T>C	0.467 (C)
SNP3	rs10742584	NG_000007.3:g.70076C>T	0.376 (C)
SNP4	rs74234654	NG_000007.3:g.70017A>T	0.310 (T)
SNP5	rs35755129	NG_000007.3:g.69994T>C	0.495 (C)
SNP6	rs11036364	NG_000007.3:g.69842T>C	0.476 (C)

The genotypes of the six SNPs in Southern China population were also downloaded from Ensembl Genome Browser (https://asia.ensembl.org/index.html), showing a heterozygosity coverage of 91.4% (96/105) in Southern Chinese (Supplementary Table S2). Chi-square test showed that the *p value* was greater than 0.05, indicating that the difference between the data from the 1000 Genomes Project (91.4%) and our individual sequencing data (93.3%) was not significant.

The six SNPs were located in region NC_000011.9:g.5247733-5249004, which is involved in nine types of β-globin gene cluster deletions in the Chinese population (Fig. 1.1-B). Based on heterozygote coverage of 93.3% (95% CI 88.57–97.14%) in normal individuals, the presence or absence of β-globin gene cluster deletion in a sample can be determined via heterozygosity analysis of the six SNPs. Detection of heterozygous SNPs in samples via genotyping enables the exclusion of large deletions in β-globin gene clusters in the SNPs location region. Conversely, if genotyping results of all six SNPs are homozygous, a 93.3% probability exists that the sample may exhibit a loss of heterozygosity at SNPs due to a large deletion in the β-globin gene cluster.

### Establishment and optimization of tetra-primer ARMS-PCR

Primer concentration optimization of tetra-primer ARMS-PCR was initially performed, using different ratios of 5:1, 2:1, 1:1, 1:2, and 1:5 for outer and inner primer concentrations, respectively. The primer concentration was then adjusted further by increasing the amount of primers for “weak” loci and decreasing the amount for “strong” loci for eliminating the uneven amplification^28^. The tetra-primer ARMS-PCR method was applied to six different SNP types (Fig. 1.3), and genotyping of the 6 different SNPs was successfully integrated into two multiplex SNPs genotyping systems (Fig. 1.4). Respective final concentrations of the primers for system A and system B were as follows: in system A, SNP1-in-F 0.04 μM, SNP1-in-R 0.2 μM, SNP3-in-F 0.04 μM, SNP3-in-R 0.2 μM, SNP5-in-F 0.2 μM, SNP5-in-R 0.12 μM, Out-F 0.2 μM, Out-R 0.2 μM, Tag-F 0.5 μM, and Tag-R 0.5 μM; In system B, SNP2-in-F 0.04 μM, SNP2-in-R 0.2 μM, SNP4-in-F 0.2 μM, SNP4-in-R 0.2 μM, SNP6-in-F 0.2 μΜ, SNP6-in-R 0.04 μM, Out-F 0.2 μM, Out-R 0.2 μM, Tag-F 0.5 μM, and Tag-R 0.5 μM.

### Performance of tetra-primer ARMS-PCR

**Figure 2:**
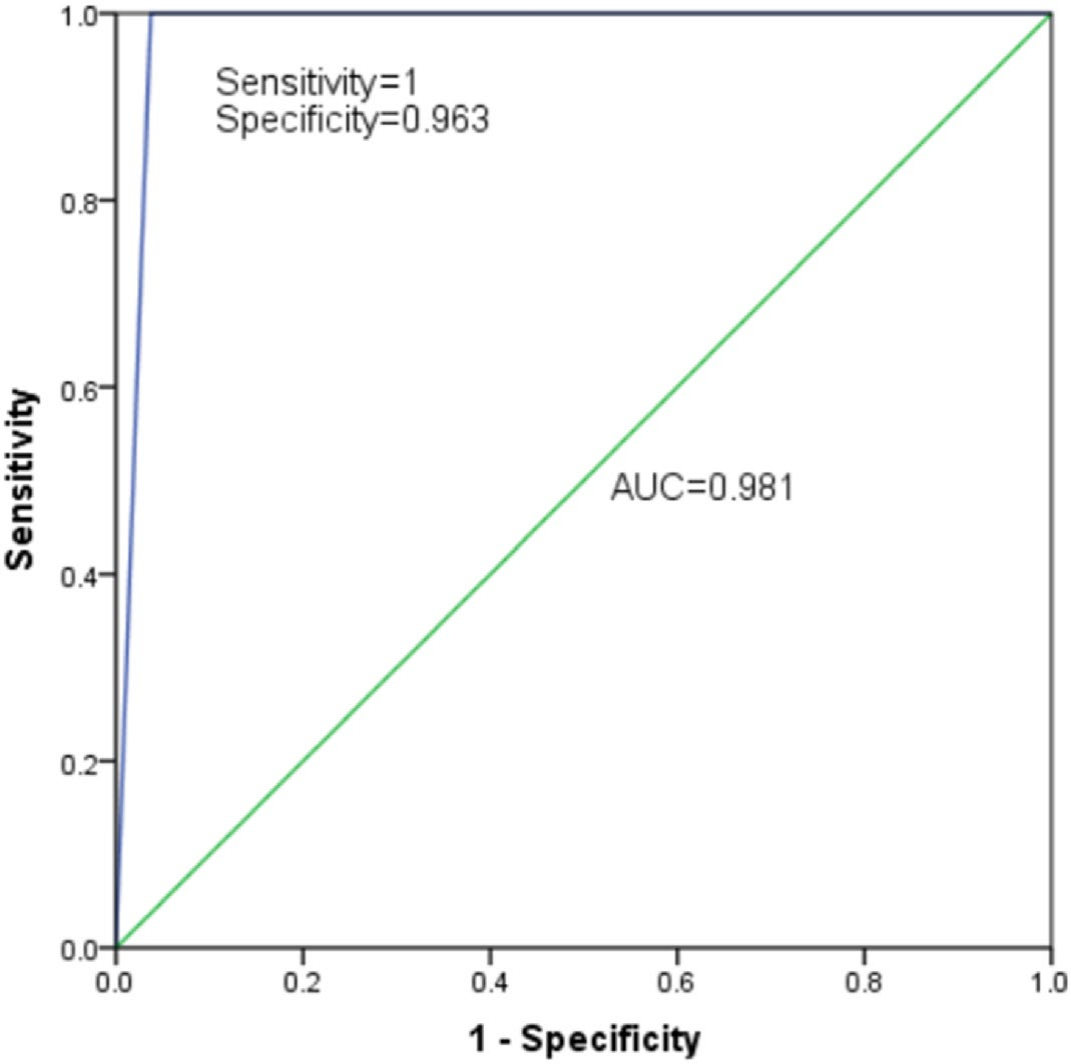
Evaluation of detection performance based on ROC curve.

40 clinical samples that suspected of having deletions in β-globin gene cluster were tested in the tetra-primer ARMS-PCR. For comparison the same sample set was also tested in the MLPA assay. The results were shown in Table 3. In tetra-primer ARMS-PCR assay, genotyping of the six SNPs showed that 27 samples had at least 1 heterozygous SNP, excluding the nine deletion mutations of beta globin gene cluster in the Chinese. All six SNPs were homozygous in 14 samples, suggesting that a large deletion of beta globin gene cluster may have occurred in these samples. The result of MLPA showed that there were 13 cases had a large deletion in β-globin gene cluster and 27 cases were normal. Therefore, there was one false positive in our study that based on SNPs analysis for the detection of β-globin gene cluster deletions using tetra-primer ARMS-PCR. The sensitivity was 100%, specificity was 96.30%, positive predictive value (PPV) was 92.86%, and negative predictive value (NPV) was 100%. A ROC curve was performed with AUC was 0.8 (Fig. 2).

**Table 3:** Clinical performance of the tetra-primer ARMS-PCR in comparison with MLPA.

Tetra-primer ARMS–PCR	MLPA		Total
	Positive	Negative	
Positive	13	1	14
Negative	0	26	26
Total	13	27	40

## Discussion

The current study developed a method for detecting β-globin gene cluster deletions based on heterozygosity analyses of SNPs. A panel of six informative SNPs obtained via bioinformatics and analyses of data from a population of 105 clinical samples, achieved a heterozygote coverage of 93.3% (95% CI 88.57–97.14%) in the normal individual. The location region in this panel of SNPs involved nine β-globin gene cluster deletion in the Chinese. For SNPs identification, a multiplex genotyping system using tetra-primer ARMS-PCR was well established and optimized. In view of low carrier rate of the β-globin gene cluster deletion in population, the efficacy of this method was validated via suspicious samples that were suspected of having large deletion in β-globin gene cluster^5^. The results of 40 clinical sample validations showed that the sensitivity, specificity, PPV and NPV of this method were 100%, 96.30%, 92.86%, and 100%, respectively.

Recent research has been directed at applying SNP analysis to detect thalassemia. For example, SNPs linked to the normal paternal α-globin allele were used for non-invasive prenatal exclusion of homozygous a^0^-thalassemia in maternal plasma^29^. This study is the first time to apply heterozygosity analysis of SNPs to detect β-globin gene cluster deletion. This was based on the principle that gene deletion leads to loss of heterozygosity at SNPs, and provided a new concept for the preliminary detection of thalassemia. The location of the six SNPs was in region NC_000011.9:g.5247733-5249004, which involved nine β-globin gene cluster deletions in the Chinese, including the two most common types, Chinese ^G^γ(^A^γδβ)^0^-Thal deletion and (SEA)-HPFH deletion. Any large deletion in this range may be detected via this method, which is suitable for detecting unknown deletions without restriction of specific deletion types. Any detection of a heterozygote SNP in the sample via genotyping enables the exclusion of a large deletion of the beta-globin gene cluster in that region. Thus, expensive and unnecessary confirmatory tests may be avoided. Conversely, if genotyping indicates that all six SNPs are homozygous, there is a 93.3% (95% CI 88.57–97.14%) probability of a deletion in the β-globin gene cluster, prompting that further laboratory examinations are needed. Currently, Gap-PCR is the most commonly used method to detect gene deletion, but it is only capable of detecting gene deletions with known breakpoints^14^. Although MLPA detects unknown breakpoints based on capillary electrophoresis technology, it is a laborious and costly method that requires significant infrastructure and skills, which is only available at specialized laboratory facilities^15^. By contrast, the method developed in this study is simple, cost-effective, low-tech, and detects deletional β-globin gene cluster disorders without requiring sophisticated equipment. Product analysis can be achieved by agarose gel electrophoresis, so the disadvantage is that it cannot be automatized. This method, which can be applied in most primary molecular laboratories, signifies a new approach to the preliminary detection of deletional thalassemia (Table 4).

**Table 4:** The characteristics of three methods.

	Gap-PCR	MLPA	New method
Time (h)	≈ 5	≈ 24	≈ 5
Operation	Simple	Complicated	Simple
Major equipment	PCR, AGE	PCR, CE	PCR, AGE
Application	Gene deletion that breakpoints are known	Both known and unknown deletional types	Detecting nine β-globin gene cluster deletions in Chinese (including other unknown deletion types in this gene region)
Cost (CNY/sample)	≈ 80	≈ 150	≈ 30

Obtaining a panel of informative SNPs, which is related to the detection of β-globin gene cluster deletion, is of critical importance to this method. The current study obtained six SNPs with incomplete linkage, all of which had MAFs greater than 0.15 ensuring at least a moderate level of informative content^30^, and three of which had MAFs greater than 0.45 (Southern China). This resulted in heterozygous coverage of the SNPs as high as 93.3% (95% CI 88.57–97.14%) of the population. The region of this six SNPs (NC_000011.9:g.5247733-5249004) was located in the common deletion region of most β-globin cluster gene deletions, involving nine large deletions of β-globin gene cluster in the Chinese. The panel is applicable to individuals from Southern China. Exploiting a large number of SNPs harbored in the genomes of most samples to increase panel size, or to screen for candidates with high MAFs, may provide a solution to the lower information content of individual SNPs^31^. Obtaining a panel of more informative SNPs via further bioinformatics and population data analyses will be conducive to improving the efficacy of this method and minimize the number of SNPs needed. Higher heterozygosity coverage rates in populations may increase the accuracy of the method. A wider distribution of SNPs may expand the application capabilities of the method and help in identifying mutation types.

An economical and effective tetra-primer ARMS-PCR method for simultaneously genotyping multiple SNPs was established. The genotyping systems of six SNPs were successfully integrated into two multiple tetra-primer ARMS-PCR reactions, which simplified experimental operation and reduced the cost of reagent consumables. The design of common outer primers in multiple systems reduced the number of primers needed. Moreover, the pair of universal primers added to the set of 5′ end primers contributed to GC percentage balance of primers and reduced the occurrence of biased and partial amplification^32^. Furthermore, temperature-switch PCR with a universal primer design may reduce the requirement for individual assay optimization and provide several technological advances for SNP genotyping, including simplified assay design and development, increased assay specificity and genotyping accuracy^33^. The study of Honardoost et al. showed that all three parameters including specificity, sensitivity and accuracy were 100% for Tetra-primer ARMS PCR method on genotyping. And comparing with Tetra-primer ARMS PCR which represented 100% agreement with sequencing method, while conventional ARMS PCR technique only showed 47.1% agreement. Similarly, our study demonstrated that the concordance between tetra-primer ARMS-PCR method and DNA sequencing was 100%, demonstrating its reliability for SNP genotyping.

**Figure 3:**
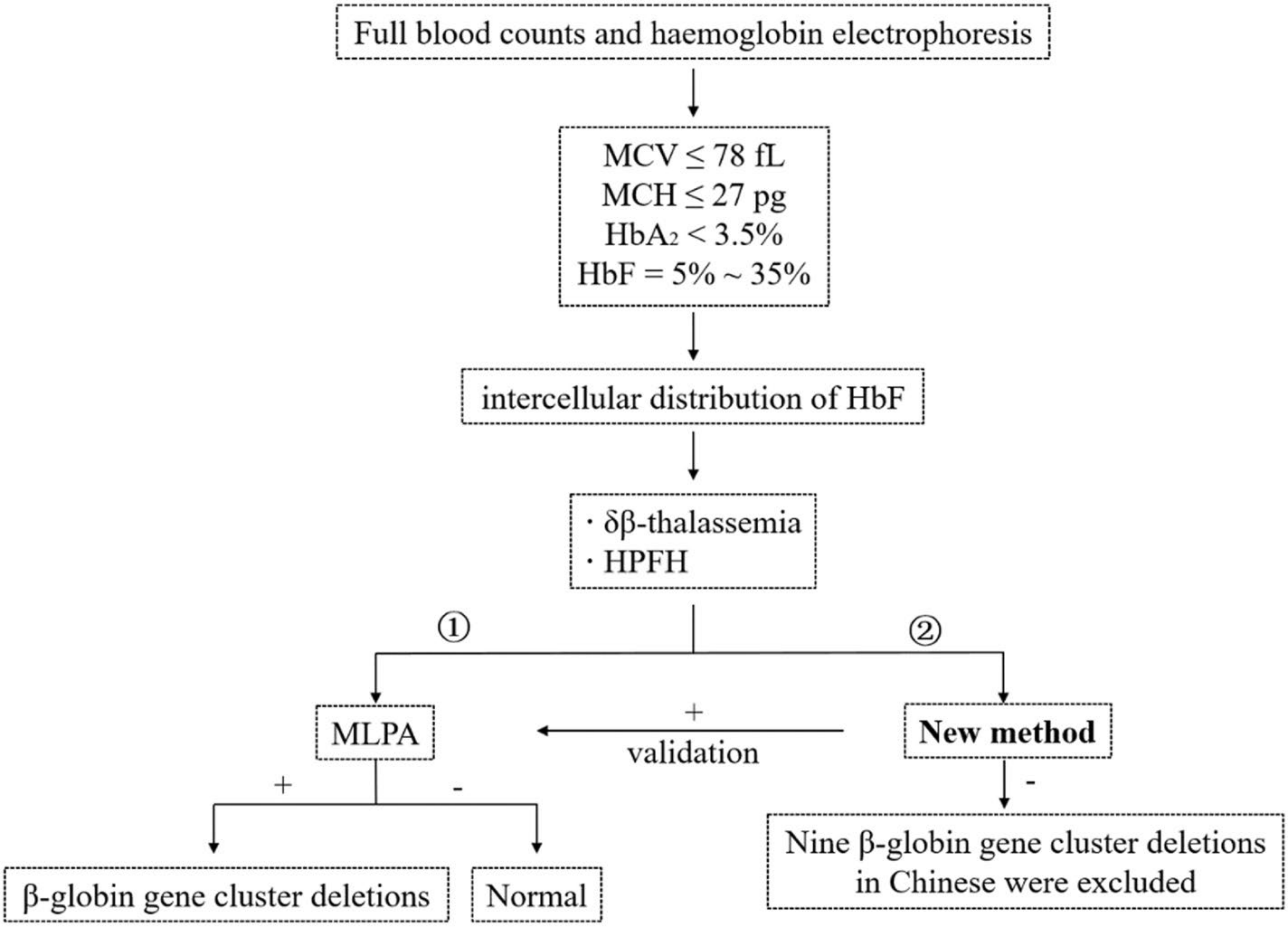
Diagnostic flowchart for identification of β-globin gene cluster deletions in this study. Choice 1: the laboratory with sufficient experimental conditions can directly carry out MLPA on suspicious samples; Choice 2: in primary laboratories lacking experimental conditions, suspicious samples can first be tested with new methods. The negative result showed that nine β-globin gene cluster deletions were excluded, and the positive result was further verified by MLPA.

In summation, the current study developed an effective, novel method for the detection of β-globin gene cluster deletion, based on SNP heterozygosity analysis. This technique provides a simple, low-tech and cost-effective detection test for deletional types of β-globin gene clusters, can be applied in most clinical molecular laboratories, especially primary laboratories with simple facilities in areas with high incidence of thalassemia. However, this method is not intended to be used as a standalone assay for making clinical decisions, the main purpose of which is to exclude large deletion in β-globin gene cluster. Thus, further laboratory testing is needed to identify specific mutation types, where results suggest the possibility of a gene deletion. The new method is suitable for use in combination with MLPA (Fig. 3). Negative samples detected by this method can exclude nine β-globin gene cluster deletions, thereby eliminating expensive diagnostic tests, and then positive samples can be further verified by MLPA method.

## Materials and methods

### Bioinformatics analysis

Based on the location of β-globin gene cluster and the breakpoint of 11 types of β-globin gene cluster deletions in Chinese (Table 1), the range of SNPs was determined to be a 32,980 bp region of the β-globin gene cluster from 5′ of *HBD* to 3′ of *SEA-HPFH del* (NC_000011.9:g.5222878-5255858). The sequence position (11_5222878-5255858) (GRCh37 version) was extracted from NCBI accordingly (https://www.ncbi.nlm.nih.gov/). SNPs data (Southern China) were downloaded from the 1000 Genomes Project (https://www.internationalgenome.org/1000-genomes-browsers). To find the most informative SNPs, we selected SNPs with a minor allele frequency (MAF) greater than 0.1 and the *p values* of the Hardy–Weinberg equilibrium tests (HWE tests) greater than 0.05. A next step to select the smallest informative set of SNPs is absence of linkage disequilibrium (LD) between the SNP positions^34^. SNP positions with r^2^ less than 0.8 were selected by HaploView. A panel of candidate SNPs was obtained.

### Population data analysis of candidate SNPs

A total of 105 clinical samples (Southern China) screened for thalassemia at the Medical Genetic Centre, Guangdong Women and Children Hospital (Guangzhou, China), were enrolled in the current study. Inclusion criteria were as follows: negative result results for both thalassemia screening (examination of red blood-cell indices and analysis of hemoglobin) and the molecular detection of thalassemia (MLPA method). MLPA reaction was performed using a SALSA MLPA P102 HBB probemix kit, according to the manufacturer’s instructions (MRC-Holland, Netherlands) (www.mlpa.com). The candidate SNP genotyping in 105 individuals was analyzed via DNA sequencing. Finally, a panel of informative SNPs that accounted for the highest proportion of samples containing heterozygous SNP in these 105 individuals was selected.

This study followed the guidelines of the Declaration of Helsinki. All experimental protocols were approved by the Ethics Committees of Guangdong Women and Children’s Hospital. The Medical Ethics Committees of Guangdong Women and Children Hospital has exempted the informed consent, because the patient information of all samples had been removed.

### Primers design

The assay consisted of 14 primers designed according to the requirements of tetra-primer ARMS-PCR^35^ (including 12 specific inner primers for the 12 alleles of six SNPs and a pair of common outer primers) and one pair of universal primers (Tag-F, Tag-R) (Table 5)^32^. Primers were synthesized by Sangon Biotech (Shanghai, China) and prepared at a concentration of 10 μM.

**Table 5:** Primers used in tetra-primer ARMS–PCR.

SNPs	Primers	Sequence (5′–3′)	Position (Chr.11.9)	Allele	Products (bp)	
SNP1	SNP1-in-F	GCGTACTAGCGTACCACGTGTCGACTGTTCATGTCATAGGAAGGGaA**T**^a,b,c^	5,247,733–5,247,754	T	SNP1-in-F + OUT-R	263
	SNP1-in-R	CAGGCCACGTTTTGTCATGCAATCTTCTAAACTGTACCCTGTTACcT**C**^a,b,c^	5,247,710–5,247,733	G	SNP1-in-R + OUT-F	1492
SNP2	SNP2-in-F	GCGTACTAGCGTACCACGTGTCGACTCAAACAGACACCATGGTGaA**T**^a,b,c^	5,248,243–5,248,263	T	SNP2-in-F + OUT-R	772
	SNP2-in-R	CAGGCCACGTTTTGTCATGCAATCTTCTCCTCAGGAGTCcG**G**^a,b,c^	5,248,226–5,248,243	C	SNP2-in-R + OUT-F	976
SNP3	SNP3-in-F	GCGTACTAGCGTACCACGTGTCGACTAGGAGAAGATATGCTTAGAcC**C**^a,b,c^	5,248,770–5,248,791	C	SNP3-in-F + OUT-R	1300
	SNP3-in-R	CAGGCCACGTTTTGTCATGCAATCTGGATGAAAACTCTACCgC**A**^a,b,c^	5,248,751–5,248,770	T	SNP3-in-R + OUT-F	451
SNP4	SNP4-in-F	GCGTACTAGCGTACCACGTGTCGACTATGTATATGTATGTGTGTATATATACACACATATATATATtT**A**^a,b,c^	5,248,829–5,248,871	A	SNP4-in-F + OUT-R	1372
	SNP4-in-R	CAGGCCACGTTTTGTCATGCAATCGATTAAAACCTTCTGGTAAGAAAAGAAAAtA**A**^a,b,c^	5,248,798–5,248,829	T	SNP4-in-R + OUT-F	396
SNP5	SNP5-in-F	GCGTACTAGCGTACCACGTGTCGACTATGCATATATATGTATATGTATGTGTGgA**T**^a,b,c^	5,248,852–5,248,881	T	SNP5-in-F + OUT-R	1390
	SNP5-in-R	CAGGCCACGTTTTGTCATGCAATCCTGGTAAGAAAAGAAAAAATATATATATATATGTGTGTATcT**G**^a,b,c^	5,248,810–5,248,852	C	SNP5-in-R + OUT-F	381
SNP6	SNP6-in-F	GCGTACTAGCGTACCACGTGTCGACTCTGCATTAAGAGGTCTCTAGTTTTTgA**T**^a,b,c^	5,249,004–5,249,031	T	SNP6-in-F + OUT-R	1537
	SNP6-in-R	CAGGCCACGTTTTGTCATGCAATCGTTTTGGGAAACAAGcG**G**^a,b,c^	5,248,987–5,249,004	C	SNP6-in-R + OUT-F	215
	Out-F	GCGTACTAGCGTACCACGTGTCGACTATTAGTCCAGGCAGAAACAGTT^a^	5,249,130–5,249,151		OUT-F + OUT-R	1660
	Out-R	CAGGCCACGTTTTGTCATGCAATCTGTTATACACAATGTTAAGGCATTAAGT^a^	5,247,542–5,247,569			
	Tag-F	GCGTACTAGCGTACCACGTGTCGACT^d^				
	Tag-R	CAGGCCACGTTTTGTCATGCAATC^d^				

^a^The universal tag sequence at the 5′ end of the primers is underlined. Tag-F denotes the 5′ of forward primer; Tag-R denotes the 5′ of reverse primer.

^b^Allele-specific nucleotides at the 3′ end are indicated by bold letters.

^c^Allele specificity is increased by introduction of a deliberate mismatch at position -3 from the 3′ terminal end of the inner primers, which is indicated by lower case letters.

^d^A pair of universal primers annealing to the 5′ portion of each chimeric primer.

### Tetra-primer ARMS-PCR

In order to simplify the operation, taking technical feasibility into account, genotyping systems of the six SNPs were integrated into two multiplex tetra-primer ARMS-PCR reactions. They were: system A (for SNP1, SNP3, SNP5 genotyping); and system B (for SNP2, SNP4, SNP6 genotyping), respectively. The proposed method was optimized in terms of primer concentration, PCR cycling conditions, and in the utilization of temperature switch PCR strategy^33^. The total volume of PCR reaction for system A and system B was 25 µL. The PCR reaction contained 12.5 μL Premix LA Taq (LA Taq Version 2.0 plus dye, Takara), 1.5 μL template DNA (50 ng/μL), an optimized concentration of each primer for system A or system B, and nuclease free water was used to bring the final volume to 25 μL. PCR amplification was performed on a Thermal Cycler (Applied Biosystems, CA). An optimized temperature switch PCR protocol, which uses two different annealing temperatures was performed as follows; initial denaturation step of 95 °C for 5 min, 20 cycles of 95 °C for 30 s, 50 °C for 30 s, 72 °C for 60 s, 15 cycles of 95 °C for 30 s, 56 °C for 30 s and 72 °C for 60 s, followed by a final extension cycle at 72 °C for 10 min, following which the preparation was cooled to 4 °C. PCR products were resolved on 1.5% agarose gel.

### Application to clinical samples

A total of 40 samples suspected to have a large deletion in the β-globin gene cluster were identified in our records from 2017 to 2019. The including criteria were hematological and molecular findings such as low blood indices (MCV < 80 fL and MCH < 27 pg), an elevated HbF levels, no known point mutation using reverse dot blot or sequencing of the whole β-globin gene, and routine α-globin genotyping (screening for -^SEA^, -α^3.7^ and -α^4.2^) was negative. The 40 samples were screened for a suspected β-globin gene deletion using tetra-primer ARMS-PCR and MLPA in parallel. The result of β-globin gene cluster identification was analyzed according to the genotypes of the panel of six SNPs, and compared with those of MLPA to evaluate the efficacy of this method for detecting β-globin gene cluster deletions. Evaluation of detection performance is based on the receiver operating characteristic (ROC) curve and the area under the curve (AUC) using SPSS software.

## Supplementary information

Supplementary Information

## Data Availability

The data that support the findings of this study are available from the corresponding author upon reasonable request.
