# Supplementary material for: Establishment and application of a novel method based on single nucleotide polymorphism analysis for detecting β-globin gene cluster deletions
Source: Sci Rep. 2020 Oct 26;10:18298. doi: 10.1038/s41598-020-75507-6 (PMC7588424; doi:10.1038/s41598-020-75507-6)
Supplement: Supplementary file 1 — Supplementary Information [file 41598_2020_75507_MOESM1_ESM.docx]

**Establishment and application of a novel method based on single nucleotide polymorphism analysis** **for** **detecting β-globin gene cluster deletions**

Siqi Hu^a,#^, Wenli Zhan^b,#^, Jicheng Wang^b^, Jia Xie^a^, Weiping Zhou^b^, Xiaohan Yang^b^, Yukun Zeng^b^, Tingting Hu^a^, Lei Duan^a^, Keyi Chen^b^, Li Du^b^, Aihua Yin^b^, Mingyong Luo^b,*^

^a^Medical Genetic Centre, Guangdong Women and Children Hospital, Guangzhou Medical University, Guangzhou, China

^b^Medical Genetic Centre, Guangdong Women and Children Hospital, Guangzhou, China

^#^These authors contributed equally to this work.

*Corresponding author: Dr. Mingyong Luo

E-mail: luo-my@163.com

| **Supplementary Table S1** The genotypes of 6 SNPs in 105 population sequencing sample in our study | | | | | | |
| --- | --- | --- | --- | --- | --- | --- |
| **Sample**  **(n=105)** | **SNP1** | **SNP2** | **SNP3** | **SNP4** | **SNP5** | **SNP6** |
|  | **rs7480526** | **rs713040** | **rs10742584** | **rs74234654** | **rs35755129** | **rs11036364** |
| 1817038 | TT | TT | TT | AA | CC | TC |
| 1817040 | TG | CC | TT | AT | TC | TC |
| 1817042 | TT | TC | CT | AA | TC | TC |
| 1817043 | TT | TC | CT | AT | TC | TC |
| 1817046 | TT | TC | CT | AT | TC | TC |
| 1817047^a^ | TT | TT | CC | AA | TT | TT |
| 1817049 | TG | TC | CT | AT | TC | TC |
| 1817050 | TG | CC | TT | TT | CC | CC |
| 1817051 | TT | TC | CT | AA | TC | TC |
| 1817052 | TG | TC | CT | AT | TC | TC |
| 1817053 | TT | TT | CC | AA | TC | TC |
| 1817054 | TT | TC | CT | AA | TC | TT |
| 1817056 | TT | TC | CC | AA | TT | TT |
| 1817057 | TT | CC | CT | AT | TC | TC |
| 1817058 | TG | TT | TT | AT | TT | TT |
| 1817061 | TG | TC | TT | AT | CC | CC |
| 1817065 | TG | TC | CC | AA | TT | TT |
| 1817066 | TT | CC | TT | AT | CC | TC |
| 1817067 | TT | TC | CT | AT | TC | TC |
| 1817068 | TG | TC | CT | AT | TC | TC |
| 1817077 | TG | TC | CT | AT | TC | CC |
| 1817078 | TT | TC | CT | AT | TC | TC |
| 1817079 | TG | TC | CT | AT | TC | TC |
| 1818002 | TG | CC | TT | AT | CC | CC |
| 1818003 | TT | TC | TT | AA | TC | TC |
| 1818007 | TT | TT | CC | AA | TC | TT |
| 1818008 | TG | TC | CT | AT | TC | TC |
| 1818009 | TT | TC | TT | AA | TC | TT |
| 1818012 | TG | CC | TT | TT | TC | TC |
| 1818013 | TT | TT | CT | AA | TT | TT |
| 1818014 | TG | TC | TT | AT | TC | TC |
| 1818019 | TG | CC | TT | AT | CC | CC |
| 1818020 | TG | TC | TT | AT | TC | TC |
| 1818022 | TT | TC | CT | AA | TC | TC |
| 1818023^a^ | TT | TT | CC | AA | TT | TT |
| 1818024 | TT | TT | CT | AT | TC | TC |
| 1818025 | TT | TC | CT | AA | TC | TC |
| 1818028 | GG | CC | TT | AT | TT | TT |
| 1818029 | TT | TC | CT | AA | TT | TT |
| 1818030 | TT | TC | CC | AA | TT | TT |
| 1818032 | TT | TC | CT | TT | CC | CC |
| 1818034 | TG | TC | CT | AT | TT | TT |
| 1818038 | TT | TC | CT | AT | TC | TC |
| 1818041 | TT | TC | CT | AA | TC | TT |
| 1818044 | TT | TC | CT | AA | TT | TC |
| 1818046 | TG | TT | TT | TT | CC | CC |
| 1818049 | TG | CC | TT | AT | CC | CC |
| 1818050 | TG | CC | TT | AT | CC | CC |
| 1818053 | TT | TC | TT | AA | TC | TC |
| 1818054 | TT | TC | CT | AA | TT | TC |
| 1818056 | TT | CC | TT | AT | TC | TC |
| 1818061^a^ | TT | TT | CC | AA | TT | TT |
| 1818067 | TT | TT | TT | AA | TC | TC |
| 1818069 | TT | TT | CT | AA | TT | TT |
| 1818073 | TT | CC | CT | AT | TC | TC |
| 1818075 | TT | TT | CT | AA | TT | TC |
| 1818092 | TT | TT | CT | AT | TC | TC |
| 1818096 | TG | TC | TT | AA | CC | TC |
| 1818098^a^ | TT | TT | CC | AA | TT | TT |
| 1818100 | TT | CC | TT | AT | TC | TC |
| 1819003 | TT | TT | CT | AA | TT | TT |
| 1819005 | TT | TT | CC | AT | TT | TT |
| 1819008 | TT | TC | CT | AT | TC | TC |
| 1819011 | TT | TC | TT | TT | TT | CC |
| 1819012 | TG | TC | CT | AA | CC | CC |
| 1819013 | TT | TC | CT | AT | TC | TC |
| 1819019 | TG | TC | CC | AA | TC | TC |
| 1819021 | TT | TC | CT | AA | TC | TT |
| 1819023 | TG | CC | CC | AA | TC | TT |
| 1819026 | TT | TC | CT | AA | TT | TT |
| 1819027 | TG | CC | TT | AA | TC | TC |
| 1819029 | TT | CC | TT | AT | TC | TC |
| 1819030 | TT | TC | CT | AA | TC | CC |
| 1819033 | TG | TC | CT | AT | TT | TT |
| 1819034 | TT | TC | CT | AT | TC | TC |
| 1819035 | TT | TC | CT | AA | TC | TC |
| 1819036 | TG | CC | TT | TT | TC | CC |
| 1819038^a^ | TT | TT | CC | AA | TT | TT |
| 1819039 | TT | TT | CT | AA | TC | TC |
| 1819048 | TT | CC | TT | AT | TC | TC |
| 1819052 | TT | CC | TT | AT | CC | CC |
| 1819054 | GG | TC | CT | AA | TC | TC |
| 1819058 | TT | TC | TT | AT | CC | CC |
| 1819063 | TT | TT | TT | AA | CC | TC |
| 12172295 | GG | TT | TT | AT | CC | TC |
| 141710829^a^ | TT | TT | TT | AA | CC | CC |
| 141714158 | TT | CC | CT | TT | CC | CC |
| 141714871 | TT | CC | TT | AA | CC | TC |
| 141718496 | TT | TT | CC | AT | TC | TC |
| 141718710 | TT | CC | TT | AA | TC | TC |
| 141720629^a^ | TT | TT | CC | AA | TT | TT |
| 141720667 | TG | CC | TT | AT | CC | CC |
| 141721787 | TT | TC | CT | AA | TT | TT |
| 141723651 | TT | TC | CT | AA | TC | TT |
| 141800268 | TT | TT | TT | AA | TC | CC |
| 141803114 | TG | TT | CT | AT | CC | CC |
| 141804467 | TT | TC | TT | AT | CC | CC |
| 141805668 | TG | TC | CT | AA | TT | TT |
| 141809426 | TT | TC | CT | AT | TC | TC |
| 141810295 | TT | TC | CT | AT | TC | TC |
| 141811742 | TT | CC | TT | TT | TC | CC |
| 141814739 | TG | TC | CT | AA | TT | TT |
| 141815617 | TG | TC | CT | AT | TC | TC |
| 141815619 | TG | CC | TT | TT | TC | TC |
| 141818560 | TT | TC | CT | AA | TC | TT |
| ^a^ Seven samples with the panel of six SNPs are homozygous;  A total of 105 samples sequencing data of 6 SNPs genotypes in our study. 98 out of 105 samples carried at least one heterozygous SNP, revealing a heterozygote coverage of 93.33%. | | | | | | |

| **Supplementary Table S2** The genotypes of 6 SNPs in Southern Chinese population from the 1000 Genomes Project | | | | | | |
| --- | --- | --- | --- | --- | --- | --- |
| **Sample**  **(n=105)** | **SNP1** | **SNP2** | **SNP3** | **SNP4** | **SNP5** | **SNP6** |
|  | **rs7480526** | **rs713040** | **rs10742584** | **rs74234654** | **rs35755129** | **rs11036364** |
| HG00403 (M) | A\|A | A\|G | A\|A | T\|T | A\|G | A\|G |
| HG00404 (F) | C\|A | G\|G | A\|G | A\|T | G\|A | G\|A |
| HG00406 (M) | A\|A | A\|A | A\|G | T\|T | A\|A | A\|A |
| HG00407 (F) | A\|A | A\|G | G\|A | T\|T | G\|G | G\|G |
| HG00409 (M) | C\|A | G\|G | A\|A | A\|T | G\|G | G\|G |
| HG00410 (F) | C\|A | G\|A | A\|G | A\|T | G\|A | G\|G |
| HG00419 (F) | A\|C | G\|G | A\|A | T\|A | G\|G | G\|G |
| HG00421 (M) | A\|C | G\|G | A\|A | T\|A | G\|G | A\|G |
| HG00422 (F) | A\|C | G\|G | A\|A | T\|A | G\|G | A\|G |
| HG00428 (F) | A\|A | G\|A | A\|G | A\|T | G\|A | G\|A |
| HG00436 (M) | A\|C | A\|G | A\|A | A\|A | A\|G | A\|G |
| HG00437 (F) | A\|A | A\|A | G\|A | T\|T | A\|A | A\|G |
| HG00442 (M) | A\|A | A\|A | A\|G | A\|T | A\|A | A\|A |
| HG00443 (F) | A\|C | A\|G | G\|A | A\|A | G\|G | G\|G |
| HG00445 (M)^a^ | A\|A | A\|A | G\|G | T\|T | A\|A | A\|A |
| HG00446 (F) | A\|A | G\|G | A\|G | T\|T | G\|A | G\|A |
| HG00448 (M) | C\|A | G\|A | A\|G | A\|T | G\|A | G\|A |
| HG00449 (F) | A\|A | A\|A | A\|G | T\|T | A\|A | A\|A |
| HG00451 (M) | A\|C | G\|G | A\|A | T\|A | G\|G | G\|G |
| HG00452 (F) | A\|C | G\|G | A\|A | A\|A | G\|G | G\|G |
| HG00457 (M) | C\|A | G\|G | G\|A | T\|T | A\|G | A\|A |
| HG00458 (F) | A\|A | G\|A | A\|G | T\|T | G\|A | G\|A |
| HG00463 (M) | A\|A | A\|A | G\|A | T\|A | A\|G | A\|G |
| HG00464 (F)^a^ | C\|C | G\|G | A\|A | A\|A | G\|G | G\|G |
| HG00472 (M) | A\|A | A\|G | A\|G | T\|T | A\|G | A\|G |
| HG00473 (F) | A\|A | A\|G | A\|A | T\|T | A\|G | A\|A |
| HG00475 (M) | A\|A | G\|A | A\|A | T\|T | G\|A | G\|A |
| HG00476 (F) | A\|A | A\|G | G\|A | T\|T | A\|G | G\|G |
| HG00478 (M) | A\|A | G\|A | A\|A | T\|T | G\|A | G\|A |
| HG00479 (F) | A\|A | G\|A | A\|G | A\|T | G\|A | G\|A |
| HG00500 (M) | C\|A | G\|A | A\|A | A\|T | G\|A | G\|A |
| HG00513 (F) | C\|A | G\|A | A\|A | A\|A | G\|A | G\|A |
| HG00524 (M) | A\|C | A\|G | A\|A | T\|A | G\|G | G\|G |
| HG00525 (F) | A\|A | G\|A | A\|A | T\|T | G\|A | G\|A |
| HG00530 (M) | A\|C | A\|G | G\|A | T\|A | A\|G | A\|A |
| HG00531 (F) | A\|A | G\|A | A\|G | T\|T | G\|A | G\|A |
| HG00533 (M) | A\|C | A\|G | G\|A | T\|A | A\|G | A\|G |
| HG00534 (F)^a^ | A\|A | A\|A | G\|G | T\|T | A\|A | A\|A |
| HG00536 (M) | A\|A | A\|A | G\|G | T\|A | A\|G | A\|G |
| HG00537 (F) | A\|C | A\|G | A\|A | T\|A | A\|G | A\|G |
| HG00542 (M) | A\|A | A\|G | G\|A | T\|A | G\|G | G\|A |
| HG00543 (F)^a^ | A\|A | A\|A | G\|G | T\|T | A\|A | A\|A |
| HG00556 (M) | A\|A | A\|G | G\|A | T\|T | A\|G | A\|G |
| HG00557 (F) | C\|C | G\|G | A\|A | A\|A | A\|G | A\|G |
| HG00559 (M)^a^ | A\|A | A\|A | G\|G | T\|T | A\|A | A\|A |
| HG00560 (F) | A\|A | G\|A | A\|A | T\|A | G\|G | G\|G |
| HG00565 (M) | A\|A | A\|A | G\|A | T\|T | A\|A | A\|A |
| HG00566 (F) | A\|A | A\|G | A\|A | A\|T | G\|G | G\|G |
| HG00580 (M) | A\|A | A\|A | G\|A | T\|T | A\|A | A\|A |
| HG00581 (F) | C\|A | G\|A | A\|A | A\|A | G\|G | G\|G |
| HG00583 (M) | A\|A | A\|A | A\|G | T\|T | A\|A | G\|A |
| HG00584 (F) | A\|A | A\|A | G\|G | A\|T | G\|A | G\|A |
| HG00589 (M) | C\|A | G\|A | A\|A | A\|T | G\|A | G\|A |
| HG00590 (F)^a^ | A\|A | A\|A | G\|G | T\|T | A\|A | A\|A |
| HG00592 (M) | A\|A | A\|G | G\|A | T\|T | A\|G | A\|G |
| HG00593 (F) | A\|A | A\|G | A\|A | T\|T | A\|G | A\|G |
| HG00595 (M) | A\|C | G\|G | A\|A | T\|A | G\|G | G\|G |
| HG00596 (F) | C\|A | G\|A | A\|G | A\|T | A\|A | A\|A |
| HG00598 (M) | A\|A | A\|A | G\|A | T\|T | A\|A | A\|A |
| HG00599 (F) | A\|A | A\|A | G\|A | T\|A | A\|G | A\|G |
| HG00607 (M) | A\|A | A\|G | G\|G | T\|T | A\|A | A\|A |
| HG00608 (F) | A\|A | G\|A | A\|G | T\|T | G\|A | A\|A |
| HG00610 (M) | A\|A | G\|A | A\|A | T\|T | G\|A | G\|A |
| HG00611 (F) | A\|A | A\|G | A\|A | T\|A | A\|A | A\|A |
| HG00613 (M) | C\|A | G\|G | A\|A | A\|A | G\|G | G\|G |
| HG00614 (F) | A\|C | A\|G | G\|A | T\|A | A\|G | A\|A |
| HG00619 (M) | A\|C | A\|G | G\|A | T\|A | A\|G | A\|G |
| HG00620 (F) | A\|A | G\|G | G\|A | T\|T | A\|G | A\|G |
| HG00622 (M) | A\|A | A\|G | G\|A | T\|T | A\|G | A\|G |
| HG00623 (F) | A\|A | A\|G | A\|A | A\|T | G\|G | G\|G |
| HG00625 (M) | A\|A | A\|A | A\|G | T\|T | A\|A | A\|A |
| HG00626 (F) | A\|A | A\|A | G\|A | T\|T | A\|G | A\|A |
| HG00628 (M) | A\|A | G\|G | A\|G | A\|T | G\|A | G\|A |
| HG00629 (F) | A\|A | G\|A | A\|A | T\|T | G\|G | G\|G |
| HG00631 (M) | A\|A | G\|G | A\|A | A\|T | G\|G | G\|G |
| HG00632 (F) | A\|A | G\|A | A\|G | T\|T | G\|A | G\|G |
| HG00634 (M) | A\|C | G\|G | A\|A | T\|A | G\|G | G\|G |
| HG00650 (M) | A\|A | A\|G | G\|G | T\|T | A\|A | A\|A |
| HG00651 (F) | A\|A | A\|A | A\|G | A\|T | G\|A | G\|A |
| HG00653 (M) | A\|A | A\|A | A\|A | T\|T | G\|A | G\|A |
| HG00654 (F) | A\|A | A\|A | G\|A | T\|A | A\|A | A\|A |
| HG00656 (M) | A\|A | G\|A | G\|A | T\|A | A\|G | A\|G |
| HG00657 (F) | A\|C | A\|G | A\|A | A\|A | A\|G | A\|G |
| HG00662 (M) | A\|A | A\|G | A\|A | T\|T | A\|G | A\|G |
| HG00663 (F)^a^ | A\|A | A\|A | G\|G | T\|T | A\|A | A\|A |
| HG00671 (M) | A\|C | G\|G | A\|A | T\|A | G\|G | G\|G |
| HG00672 (F) | A\|C | A\|G | G\|A | T\|A | A\|G | A\|G |
| HG00674 (M) | A\|A | G\|A | A\|G | T\|T | G\|G | G\|G |
| HG00675 (F) | A\|A | G\|A | A\|A | T\|T | G\|A | G\|A |
| HG00683 (M) | A\|A | A\|G | G\|G | T\|T | A\|A | A\|A |
| HG00684 (F) | A\|A | A\|G | G\|A | T\|T | A\|G | A\|G |
| HG00689 (M) | A\|A | G\|A | A\|G | A\|T | G\|A | G\|A |
| HG00690 (F) | A\|A | A\|A | A\|A | A\|T | A\|A | A\|G |
| HG00692 (M) | A\|C | G\|G | G\|A | T\|A | A\|G | A\|G |
| HG00693 (F) | A\|A | G\|A | G\|A | T\|T | A\|G | A\|G |
| HG00698 (M) | C\|A | G\|G | A\|A | A\|T | G\|G | G\|G |
| HG00699 (F) | C\|A | G\|A | A\|G | A\|T | G\|A | G\|A |
| HG00701 (M) | A\|A | G\|A | A\|G | T\|T | G\|A | G\|A |
| HG00704 (M) | A\|A | G\|G | G\|A | T\|T | A\|G | A\|A |
| HG00705 (F) | A\|A | G\|A | G\|A | T\|T | A\|G | A\|A |
| HG00707 (M) | A\|A | A\|A | A\|G | A\|T | A\|A | G\|A |
| HG00708 (F) | A\|C | G\|G | A\|A | A\|A | G\|G | G\|G |
| HG00717 (F)^a^ | A\|A | A\|A | G\|G | T\|T | A\|A | A\|A |
| HG00728 (M) | A\|C | A\|G | G\|A | T\|A | A\|G | A\|G |
| HG00729 (F)^a^ | A\|A | A\|A | G\|G | T\|T | A\|A | A\|A |
| ^a^ Nine samples with the panel of six SNPs are homozygous;  A total of 105 data was downloaded from Ensembl Genome Browser *(http://asia.ensembl.org/index.html)* (Southern Chinese population). 96 out of 105 samples carried at least one heterozygous SNP, revealing a heterozygote coverage of 91.43%. | | | | | | |
